# Cross-Scale Convergence in Epigenetic Gene Regulation: A Perspective on Functional Enrichment Analytics for Cancer

**DOI:** 10.3390/cimb48090915

**Published:** 2026-09-07

**Authors:** Adam G. Marsh, Ashley S. Doane

**Affiliations:** 1 Center for Computational Biology and Bioinformatics, University of Delaware, Newark, DE 19716, USA; 2 SignPost Cancer Analytics, Inc., Kingston, ON P.O. Box 26, Canada; ashleydoane@signpostcanceranalytics.com; 3 Institute for Computational Biomedicine, Weill Cornell Medicine, New York, NY 10065, USA

**Keywords:** epigenetics, cross-scale regulation, DNA methylation, chromatin remodeling, Polycomb repressive complex, bistability, topologically associating domains, cancer epigenomics, multi-omic data integration, functional enrichment analysis

## Abstract

Epigenetic regulation of gene expression is studied at three physical scales: micro: DNA sequence-level methylation/demethylation; meso: nucleosome occupancy and remodeling; and macro: chromosomal domain silencing by Polycomb complexes, heterochromatin, and topologically associating domain (TAD) boundaries. The challenge to fully understand epigenetic gene regulation patterns is that these scales are not independent. Their influence overlaps and they share a recurring architectural theme across scales of a targeted molecular pattern followed by cooperative, feedback-driven, spatially bounded spreading. We argue here that disruption of this shared architecture at any one scale is independently sufficient to tip a bistable silencing domain into an oncogenic state. This paper discusses how such a cross-scale architectural rule set has concrete implications (yet underexploited) for computational cancer epigenomics, e.g., functional enrichment analyses generally focus on epigenetic features at one scale as an independent line of evidence, ignoring corroborating signals that could be reinforced by underlying hierarchical levels. This paper outlines options for functional enrichment statistics that combine multiple corroborating molecular features within a scale and corroborating evidence across scales into composite confidence scores calibrated against an empirical null that preserves correlations between assays. We propose benchmarking this approach against conventional single-feature enrichment in matched multi-omic cancer datasets as a direct test of the model.

## 1. Introduction

Epigenetic gene regulation is usually studied one scale at a time, with a DNA methylation paper here, a chromatin-remodeling paper there, a Polycomb-domain paper elsewhere, as though these were separate research programs rather than facets of one multi-scale phenomenon. At the **micro** scale, single enzymes read local DNA sequence context to decide where to place or remove a methyl group. At the **meso** scale, nucleosomes are positioned, evicted, or stabilized by remodeling complexes that read histone post-translational modifications (PTMs). At the **macro** scale, kilobases-to-megabases of chromatin are folded into insulated domains and coated with repressive chemical modifications that silence entire gene neighborhoods.

This perspective argues that these three scales are not coincidentally related, but they instead share one recurring mechanistic architecture, and that this shared architecture has a direct, underexploited implication for computational cancer epigenomics: functional enrichment analyses that treat each scale, and each molecular feature within a scale, as an independent line of evidence are ignoring corroborative, cross-scale signals that are established by the hierarchical organization in human genomes. First, the micro-, meso-, and macro-scale literature is briefly summarized to establish shared structural or architectural rule-sets that promote a hierarchical organization. Second, the cross-scale commonalities that follow from that organization are described. Finally, this conception is presented in the context of an integrated bistable-switch model to identify concrete options for functional enrichment statistics that draw on multiple corroborating features within and across scales to strengthen predictive confidence in a given cellular-function or pathway call.

## 2. Epigenetic Scales

### 2.1. Micro Scale: DNA Sequence-Level Mechanisms

At DNA molecular sequence scales, DNA methyltransferases and demethylases do not act indiscriminately on every cytosine-guanine (CpG) dinucleotide. They read the one or two base pairs flanking the target CpG and their catalytic activity is impacted accordingly, sometimes by more than 100-fold.

The de novo methyltransferases DNMT3A and DNMT3B have distinct, structurally encoded flanking-sequence preferences. DNMT3A favors a CG(C/T) motif and is most sensitive to the bases two positions upstream and downstream of the CpG, while DNMT3B favors a CG(G/A) motif, in particular, a guanine immediately $3^{\prime }$ of the CpG (a “CpGpG” context), and it shows 12- to 30-fold higher activity at CpG than at CpA or CpT sites [1]. Structural work has resolved the physical basis for this: in DNMT3B, residues N779 and K777 in the target-recognition-domain (TRD) loop make direct hydrogen-bond and van der Waals contacts with the +1 flanking guanine, with K777 additionally sensing the shape and polarity of the major groove at that position [2]. The stimulatory cofactor DNMT3L does not rewrite this sequence-preference ranking; instead it selectively boosts methylation at otherwise poorly methylated contexts, flattening the sequence bias and producing the more uniform genome-wide methylation seen in vivo [3]. Basically, the “writer” enzyme’s catalytic geometry is a direct reader of local DNA sequence.

The demethylation machinery shows an analogous logic. TET enzymes oxidize 5-methylcytosine in a pronounced, sequence-dependent manner, with up to 250-fold differences in activity in vitro depending on the flanking bases; the single most favorable context is the CACGTG E-box hexamer, the canonical binding motif for basic helix-loop-helix (bHLH) and basic-leucine-zipper (bZIP) transcription factors [4]. Crystal structures show this arises not from direct DNA base-readout contacts (as in DNMT3B) but from the flanking sequence indirectly reshaping the enzyme’s conformation via intra-substrate interactions [4]. This is an important convergence where the sequence context most favorable for active demethylation is literally a transcription-factor recognition motif, wiring TET targeting to the same sequence code that transcription factors themselves read [4,5].

That wiring becomes explicit and functional in a genome-wide screen that identified eight transcription factors (RUNX3, GATA2, CEBPB, MAFB, NR4A2, MYOD1, CEBPA, and TBX5) that actively induce DNA demethylation centered on their own binding sites [6]. Importantly, this demethylation is not a broad, genome-wide erasure. It extends only about 100–320 bp from the motif, and it preferentially targets CpGs that started out hypermethylated, i.e., methylation-state-dependent and sequence/motif-directed local editing rather than random or unbounded demethylation [6]. The micro-scale picture that emerges shows enzymes with intrinsic, structurally encoded sequence specificity acting at, and only locally around, defined DNA motifs, with methyltransferases, demethylases, and transcription factors all reading overlapping or identical sequence codes.

### 2.2. Meso Scale: Nucleosome Occupancy and Positioning

At the next hierarchical level, the unit of regulation is the nucleosome. The operational question here becomes not “which sequence is modified” but “which 147 bp segment of DNA is wrapped, exposed, or evicted.”

ATP-dependent chromatin remodelers are the principal engines of nucleosome occupancy. The ISWI family achieves precise, regularly spaced nucleosome arrays through a conserved ATPase translocase “motor,” whose targeting specificity and degree of DNA translocation are tuned by accessory regulatory subunits. Here, a single catalytic mechanism has diversified by modular, swappable control subunits [7]. The resulting structural state is itself gated by “reading” histone PTMs with remodeler subunits that carry bromodomains (recognize acetylated lysines) and PHD fingers (recognize methylated lysines), including a basic-patch motif on the histone H4 tail that acts as an activation signal. In this class, chromodomains also represent an important third major reader family recognizing methylated lysines. Thus, the histone modification state of a nucleosome directly shapes whether and how it is remodeled [7]. A more recent, comprehensive review frames this as “energy-driven genome regulation” where remodelers convert the chemical energy of ATP hydrolysis into positional information, reading nucleosome and DNA features to decide the wrapping status of nucleosomes [8].

Nucleosome stability, not just position, is then separately tuned by histone variant incorporation. Nucleosomes containing the replication-independent variant H3.3 are markedly more salt-sensitive (less stable) than canonical H3.1/H3.2 nucleosomes, and nucleosomes carrying both H3.3 and the variant H2A.Z are the least stable combination of all. Although this instability is unusual, it is not incidental but functional since this double-variant destabilized nucleosome type is preferentially enriched at promoters and enhancers of actively transcribed genes [9]. In other words, the cell deliberately builds unstable nucleosomes at exactly the loci that need to remain dynamically accessible.

Finally, meso-scale remodeling is directly coupled to macro-scale silencing machinery. The loss of the SWI/SNF (BAF) complex subunit BAF47/SMARCB1 prevents BAF from evicting Polycomb Repressive Complex 2 (PRC2) and its H3K27me3 mark from the *CDKN2A*/Ink4a tumor-suppressor locus, permitting inappropriate silencing and cellular transformation [10]. BAF/SWI-SNF complexes are themselves mutated, collectively, in more than 20% of human cancers across a broad range of tumor types, making them the single most frequently mutated chromatin-regulatory complex family in cancers [10]. The meso-scale dynamics are defined by remodeler motor activity, tuned by PTM-reading accessory domains and by variant-driven nucleosome (in)stability, which determines local accessibility. Thus, this layer is a direct gatekeeper for whether macro-scale repressive domains can be established or removed.

### 2.3. Macro Scale: Chromosomal Domain Silencing

At the upper scale, individual nucleosome states are aggregated into stable, heritable, kilobase-to-megabase domains of heterochromatin, gating the expression of “silenced genes.” This Polycomb machinery system impacts processes such as epigenetic cell memory systems, stem cell regulation, environmental acclimation responses, and disease onset or progression when dysregulation is deleterious (i.e., cancer).

Polycomb machinery functions are the paradigm model for epigenetic macro-scale processes. Polycomb Repressive Complex 2 (PRC2) deposits H3K27me3 and spreads it across a domain via allosteric self-stimulation; this spreading is reinforced by an explicit positive-feedback loop in which H2AK119ub, deposited by PRC1, recruits PRC2 and deposits more H3K27me3. In turn, this helps to recruit more PRC1, which forms a self-reinforcing loop to convert a nucleation event into a stable, propagated silenced chromosomal domain [11]. This is a textbook case of cooperativity/positive feedback as the physical molecular process that promotes domain-scale spreading. Polycomb dysregulation is mechanistically implicated in cancer through two distinct routes: (i) aberrant silencing of tumor-suppressor genes, and (ii) inappropriate activation of developmental/stemness programs that should otherwise be shut off in differentiated cells [11,12]. Gain-of-function mutations in the PRC2 catalytic subunit EZH2 (e.g., at Y641) drive H3K27 hypermethylation and oncogenic transformation in lymphoma, while, conversely, PRC2 loss-of-function can be oncogenic in other contexts (e.g., malignant peripheral nerve sheath tumors) by de-repressing growth or stemness programs, which underscores that it is dysregulation of domain-silencing machinery, and not silencing or its loss alone, that is oncogenic [12].

A second, independent macro-scale silencing mechanism is the formation of Large Organized Chromatin K9-modifications (LOCKs). These structures are megabase-scale H3K9me2 domains whose genomic coverage increases dramatically with differentiation, from roughly 4% of the genome in embryonic stem cells to ∼31% in differentiated ES-cell derivatives and up to ∼46% in liver tissue. This progression demonstrates that macro-domain heterochromatinization is in itself a developmentally regulated, expanding process, not a static genomic feature [13].

Overlaid on both of these epigenetic-based silencing systems is the three-dimensional folding of chromatin into Topologically Associating Domains (TADs). These are self-interacting neighborhoods defined by CTCF/cohesin-anchored boundaries that “insulate” genes inside a domain from regulatory elements outside of that domain [14]. This architecture is generated mechanistically by loop extrusion, in which cohesin processively enlarges a chromatin loop until halted at convergent CTCF binding sites that act as an asymmetric, DNA-tension-dependent barrier [15,16]. Even across cancer types, this megabase-scale architecture (A/B compartments and TADs) is largely conserved, while finer-scale enhancer–promoter looping varies substantially between tumor types. This variability suggests TAD-level architecture is a relatively stable scaffold upon which finer tuning of regulatory responses (and pathological variation) is built [17]. Thus, the macro-scale structural frame is defined by cooperative, feedback-driven spreading of repressive histone marks (Polycomb, LOCKs) that result in domain-scale silencing that is physically demarcated by an independent, CTCF/cohesin-based, 3D-folding layer that sets the length of the domain’s spatial extent.

### 2.4. Inherent Limitations of Solo Scale Silos

The concept of mirco-, meso- and macro-scale genomics is evident in how published studies almost exclusively focus on a single level. This statement is not critical of existing work because these scales also represent “silos” of expertise, laboratory infrastructure, national funding programs, graduate training programs, etc. We are approaching this proposal idea not from the perspective that these studies are flawed and need to be fixed, but from the perspective of asking whether there are missed opportunities that could be useful in approaching epigenomics across such genome scales. The three studies below are recent, large, and important works within their fields. They are all methodologically sound, yet each is limited to some degree in its potential discovery solely as a result of a single-scale design rather than any aspect related to the execution of the work. Again, these examples are excellent studies, but each could have had more impact by adding a cross-scale dimension to their analyses.

Micro scale: Pan-cancer DNA methylation. Large-scale analyses of tumor methylomes across dozens of cancer types have identified recurrent promoter CpG-island hypermethylation at tumor-suppressor loci. These results are important and have been used to identify driver genes and enriched pathways that are epigentically silenced [18]. The inference these designs cannot make is functionally important: CpG-island hypermethylation in cancer preferentially targets promoters that were already Polycomb-marked and transcriptionally repressed in the corresponding progenitor state [19,20,21]. Without the meso- and macro-scale state of those loci measured on the same tumors, a hypermethylation event that silences a previously active gene is statistically indistinguishable from one that consolidates a pre-existing repressed state. In the first instance, a candidate driver lesion is identified, while in the second a passenger or hitchhiker consequence of the cell’s lineage is cataloged. The analytical problem here is that a pathway enriched for the second passive occurrence will be reported with the same confidence as one enriched for the first actual driver of the oncogenic state [22]. This confounded dichotomy is the best-documented systematic bias in cancer methylation analyses, and enrichment studies conducted solely on a single-scale have no mechanism for correcting for that bias.

Meso scale: Pan-cancer chromatin accessibility. The pan-cancer ATAC-seq atlas established accessible regulatory landscapes specific to cancer-type across hundreds of primary tumors and inferred candidate enhancer–gene links and transcription-factor drivers [23]. The primary analysis is accessibility-anchored sequencing. Differential accessibility at a locus is therefore not separable from a compartment-level A-to-B switch (up-scale rearrangement) affecting the entire surrounding neighborhood with only meso-scale data. Likewise, the loss of accessibility at a promoter via ATAC-seq data does not discriminate between whether down-scale methylation events are likely drivers or hitchhikers. The consequence for functional enrichment is specifically a higher false positive rate where a pathway whose genes cluster in the genome (like many developmental and immune pathways) would be nominated as an oncogenic driver on just the strength of what is effectively a single meso compartment change naive of whether or not a proximal switch was triggered by coordinated micro-scale epigenetic silencing of a set of gene promoters.

Macro-scale: Cancer 3D genome architecture. Systematic analyses of TAD organization and structural variation across tumor types have shown that boundary disruption is recurrent and associated with expression change at neighboring genes [24]. What is generally not measured on the same specimens is the micro- and meso-scale state at those boundaries. Since CTCF occupancy is methylation-sensitive [25], a lost boundary may reflect a structural deletion, a CTCF-site hypermethylation event, or a change in cohesin loading. These three mechanistically distinct lesions have distinct therapeutic implications, indistinguishable from the contact map alone. Functional enrichment run on boundary-disrupted gene neighborhoods will aggregate all three mechanisms as one overall effect.

There is value of integration across scales. As an example, mutations in IDH produce an oncometabolite that inhibits TET causing hypermethylation at CTCF binding sites, disrupts TAD insulation, and permits an enhancer to activate *PDGFRA*. This causal chain traces explicitly from micro to macro scales in the same tumor system [25,26]. This is a concrete result that no single-scale design could have produced. This result was not obtained by a more sensitive methylation analysis, nor by a higher-resolution contact map, but by asking what a micro-scale lesion does to a macro-scale structure. Similarly, the demonstration that loss of the BAF subunit SMARCB1 prevents eviction of PRC2 from *CDKN2A* connects a meso-scale complex to a macro-scale silencing outcome [10], and it is likewise unavailable from either scale alone.

There is a common limitation in all three cases. In the three single-scale studies above, the enrichment step treats one feature type as the complete evidence for a gene’s role in tumor growth or suppression. In functional enrichment analyses, each pathway identified as significant therefore inherits the confounding factors that are specific to that data channel: cell-of-origin chromatin state and tumor purity for methylation, peak-calling sensitivity and compartment structure for accessibility, resolution and coverage for contact maps. Each solo data channel has no independent channel available to discriminate direct confounding functional variance from indirect nuisance factor variance. The logical assertion here is simply that corroboration is needed in these analyses and that data currently exist (or are methodologically accessible) to separate between driver lesions from channel-specific artifacts by using datasets with information distributed across scales.

### 2.5. Multiple Genomic Scales Exist: Three Is Not a Magic Number

The partition into micro, meso, and macro scales is a descriptive convenience. These three levels have well-developed and largely separate the literature, and they span the range of length scales over which epigenetic regulation is conventionally studied. It is not a claim that chromatin has exactly three levels of organization, and nothing in the argument developed here is dependent upon utilizing only three grouping levels.

What the argument proposal here does require is that a level satisfy four conditions. It must have a characteristic length scale separable from those of adjacent levels. It must exhibit dynamics distinguishable from those levels so that it is not merely a finer description of the same object (impacted by the exact same variance sources). It must instantiate the shared architectural logic developed in Section 3 with targeted readout, cooperative reinforcement, and spatially bounded spreading. Lastly, it must be accessible through a measurement channel whose error structure differs from those of the other levels. In this proposal, micro, meso, and macro satisfy these conditions, but these are not the only possible scale divisions.

Nothing presented in this proposal runs contrary to any study using a different number of level designations for their research goals. A clear fourth candidate for a scale could span between the nucleosome and the insulated domain to describe a specific, functional contact between a distal regulatory element and a promoter. This length scale, roughly one to a hundred kilobases, is bounded below by nucleosome-level accessibility and above by domain-level insulation. Its dynamics are distinguishable from those of TADs, which are stable, largely cell-type-invariant, and generated by loop extrusion halted at CTCF sites [15,16]. Because enhancer–promoter contacts within them are labile, and not straightforwardly dependent on that machinery [27,28,29,30], acute cohesin depletion eliminates TADs and CTCF loops while leaving much enhancer-driven transcription active as well as many fine-scale contacts intact [31,32,33].

In this case, the targeted readout is a reader module recognizing an element’s mark combination (a chromodomain reading H3K27me3 and a bromodomain reading H3K27ac [7,34]) with sequence-specific factors stabilizing the contact. The cooperative step is mutual reinforcement between contact formation and coactivator or corepressor accumulation [35,36]. The bounded extent is the contact itself as a defined element-to-promoter pairing rather than a spreading front, which makes this level more gene-specific than any structural level above it. Whether its measurement channel is sufficiently independent of nucleosome-level accessibility to satisfy the fourth condition is an empirical question that is more tractable than attempting to ask how many levels chromatin“really” has. The three levels developed in this perspective are accordingly the foundation of this proposal, but these three levels do not describe a boundary on the application of the statistical framework that is proposed here.

## 3. Cross-Scale Commonalities

The features reviewed in Section 2 and highlighted in Table 1 have operationally similar activities and indicate that the epigenetic-driven structural determinants across scales are a single recurring architectural rule set and not three unrelated mechanisms. There are five commonalities identified here as the core of this idea. These five processes are not merely functional descriptions but instead define concrete feature types: (i) molecular sequence/pattern context, (ii) PTM co-occurrence, (iii) epigenetic mark inheritance, (iv) phase-separated compaction, and (v) boundary integrity to control spreading extent.

### 3.1. Targeted Readout Drive Locally Bounded Spreading

At every scale, a “reader” component recognizes a specific, narrow input: at the micro scale, a flanking DNA sequence (DNMT3A/B, TET) [1,2,4]; at the meso scale, a histone PTM read by a recognition module carried on a remodeler subunit, with bromodomains recognizing acetylated lysines and PHD fingers recognizing methylated lysines [7]; and at the macro scale, a pre-existing repressive mark read through either chromodomains or PRC1/PRC2 complexes. Polycomb recognition is the important event at this upper scale because here the reading event is also the propagation step with CBX subunits of canonical PRC1 reading H3K27me3 through the aromatic cage of a chromodomain [34,37,38], while PRC2 reads the H2AK119ub that PRC1 deposits [11]. Each complex therefore recognizes the mark left by the other, and it is this reciprocal recognition that closes a self-reinforcing loop and converts a nucleation event into a heritable propagated domain. These recognition events spread outward to different boundary extents: ∼100–300 bp for TF-directed demethylation [6], bounded to the local nucleosome cluster engaged by the remodeler so it does not propagate without limit along the fiber [7,8], and at the macro scale to a domain bounded by CTCF/cohesin insulators [14,17].

### 3.2. Cooperativity and Positive Feedback Drives Spreading

The PRC1–PRC2 mutual-recruitment loop [11] is the clearest macro-scale example, and the same logic has been formalized at the level of individual nucleosomes. In a classic theoretical and experimental analysis of epigenetic cell memory, Dodd et al. showed that robust bistability of a chromatin modification state requires cooperativity of reactions in which more than one already-modified nucleosome contributes to modifying a target [39].

The geometry of this cooperativity is often simplified to linear sequence models but in fact entails more complex spatial interactions. Dodd et al. found that spreading restricted to immediate linear neighbors does not produce robust bistability. This property emerges only when nucleosomes distant in the linear sequence contribute to modifying a target, i.e., when recruitment is mediated by three-dimensional proximity rather than by adjacency along the fiber [39]. Spreading is therefore better described as contact-guided than as progressive along a chain. Nucleosomes are acted on in small clusters rather than one at a time, and nucleosomes interposed in the linear sequence can be skipped while more distant, spatially proximate nucleosomes are modified.

Chromatin in the nucleus is now viewed as an irregular 30 nm fiber of variable-diameter chain [40] organized into heterogeneous nucleosome groups [41]. It is this heterogeneity that supplies exactly the structural variance that local clustering requires of a cluster-wise mechanism. Repressive-domain formation initiates at discrete nucleation sites and propagates through looping contacts, producing domains that are contiguous in contact space but are not necessarily modified in linear space [42]. Propagation also requires more than the mark itself with dependencies on sequence-specific recruitment to sustain a Polycomb-repressed state [43] as in the inheritance of H3K27me3 that is causally required to maintain silenced states in some HOX genes (*Drosophila*, [44]). Thus the read-write propagation operates alongside, rather than in place of, targeted recruitment mechanisms with direct measurements of spreading kinetics in living cells being consistent with this concept [45].

The developing picture is therefore one with a contact-domain-shaped, cluster-wise process in which linear distance is a proxy for, but not the determinant of, the probability of modification. Mechanistically, this explains how a repressive domain can be interrupted by an active, contact-excluded element without collapsing, which is a pattern conspicuous at Polycomb-regulated developmental loci such as the HOX clusters (Section 2.4). Importantly, the phase-separation account (Section 3.3) is a three-dimensional mechanism by construction where a condensate concentrates modifying enzymes within a restricted spatial volume, and every nucleosome recruited into that volume is a substrate irrespective of its linear position. Analytically, the mechanism dictates that the aggregation neighborhood for a domain-level score should be defined by measured contacts rather than by a fixed window of linear genomic distance.

### 3.3. Biophysical Phase Separation

A biochemical feedback loop still needs a physical mechanism to compact and maintain a multi-kilobase domain in the nucleus. HP1 proteins undergo liquid-liquid phase separation (LLPS) in vitro and form liquid-like, coalescing nuclear foci during heterochromatin domain formation in vivo [46,47]. HP1 binding measurably increases the accessibility and dynamics of otherwise buried histone-core residues, and it is this conformational “opening” of the nucleosome core that promotes phase separation [48]. Essentially, with nucleosome expansion and increased nucleosome–nucleosome multivalency, a phase-separated, liquid state becomes more persistent (favored conformation) [49]. Phase separation therefore offers a biophysical account of how a self-reinforcing feedback loop is converted into an actual, membrane-less, spatially compartmentalized silenced domain, bridging meso-scale nucleosome dynamics directly to macro-scale domain compaction. Phase separation is also the physical reason that spreading is contact-guided rather than linear (Section 3.2) because a condensate defines a spatial neighborhood of substrate nucleosomes, not a sequential series of proceeding neighbors.

### 3.4. Conserved Sequence/Mark-Directed Targeting

The specific alphabet differs by scale: DNA bases at the micro scale, histone PTMs at the meso scale, and pre-existing chromatin marks at the macro scale. Regardless, the logic of “a reader protein/domain converts a specific input pattern into a targeted catalytic or structural output” is recurrent at all three scales. All levels of molecular patterns can be described here as a “code”, used in the sense of the histone-code hypothesis, in which covalent histone modifications form a combinatorial marking system that is read by effector proteins and translated into a downstream chromatin state changes [50,51]. This combinatorial mapping “code” is not a linear logic sequence and it has been shown that these marks are a degenerate, context-dependent, heavily co-occurring signal rather than a cipher with a one-to-one correspondence reaction [52]. What is conserved is the reader–writer coupling, not the strictness of the mapping.

Another dimension of variability is imposed by the fact that the implementations of that coupling (code to reaction) are strikingly heterogeneous. DNMT3B reads flanking bases by direct hydrogen-bond and van der Waals contact [1,2], whereas TET enzymes read their preferred E-box context indirectly, through flanking sequence reshaping the enzyme–substrate conformation [4]. Here are just two physically opposite readout strategies on the same alphabet, both terminating in a catalytic rate that is a steep function of local sequence. At the meso scale the architecture becomes explicitly modular with a conserved ATPase motor coupled to swappable bromodomain and PHD-finger reader modules [7,8]. At the macro scale the input is a pre-existing mark, read by PRC2 and PRC1 in mutual recruitment [11] or by HP1, whose output is biophysical rather than catalytic [46,48].

Importantly, a disruption within any single layer can converge on the same oncogenic endpoints. Loss of BAF47 blocks meso-scale eviction of a macro-scale Polycomb mark from a tumor-suppressor locus [10]; IDH mutations generate an oncometabolite that inhibits TET (a micro-scale enzyme) [26], causing hypermethylation at CTCF sites that disrupts macro-scale TAD insulation and permits oncogene activation [25]; and direct excision of a CTCF-bound insulator (a macro-scale structural element) de-represses the *TAL1*/*LMO2* proto-oncogenes in T-cell leukemia [53]. Three entirely different molecular starting points converge on the identical oncogenic logic that arises when a stable locally bounded, cooperative silencing/insulating mechanism is lost and the regulatory controls over the elements within that domain are suddenly flipped.

## 4. Integrated Model for Cancer Onset and Progression

### 4.1. The Integrated Idea

**Figure 1:**
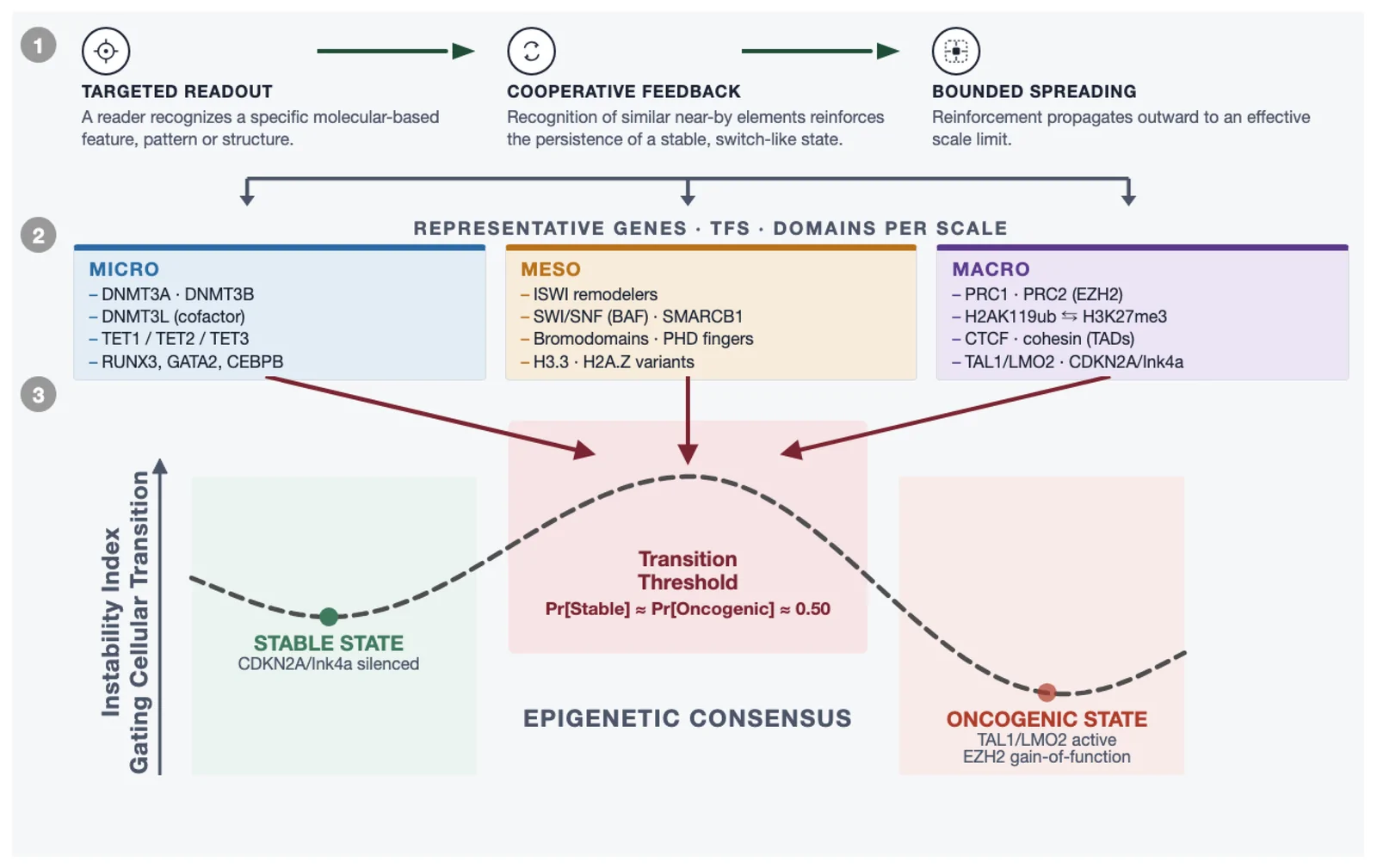
Schematic cross-scale synthesis of shared epigenetic architecture. Level 1 depicts the three-step logic shared across all scales: targeted-readout, cooperative-feedback, and bounded-spreading steps. Level 2 provides representative micro-, meso-, and macro-scale features. Level 3 recasts the bistability argument as an Instability Index curve along a binomial Epigenetic-Consensus axis.

Integrating these commonalities suggests a single, cross-scale mechanistic model for how epigenetic dysregulation drives cancer, rather than three disconnected “hits.” Normal cellular identity is maintained by a nested hierarchy of locally targeted, cooperatively reinforced, spatially bounded silencing that spans all three scales [54]. Because feedback and phase-separation-driven spreading are inherently threshold/bistable phenomena [39], this system does not require simultaneous failure at all three scales to flip a domain’s silencing state. A single lesion at any scale, whether a sequence-reading enzyme mutation, a remodeler-subunit loss, or a structural deletion of a domain boundary [10,24,25,53], can be sufficient to push a previously stable domain past its switching threshold (Figure 1). The convergent examples of Section 3.4 establish that lesions originating at different scales can reach the same endpoint; however, whether any one is sufficient in isolation, and independent of the other scales, is an empirical question that the proposed model approach makes testable.

The same micro-scale sequence-reading machinery that normally protects CpG-island promoters from methylation can be corrupted to become the agent of their aberrant hypermethylation and tumor-suppressor silencing in cancer, a pattern documented at pan-cancer scale across thousands of tumors [18,22]. Because this crossing recruits the same feedback and phase-separation properties that stabilize normal identity, the resulting state is heritable and increasingly resistant to reversal. This results in a “punctuated equilibrium” escape whose effect is like a ratchet progression towards oncogenic dysregulation. The bistable effect acts primarily by corrupting the targeting and feedback layers within a broadly conserved structural scaffold [17], rather than by wholesale rearrangement of 3D genome architecture (Figure 1).

In Figure 1, a transition threshold state recasts the bistability argument as an instability curve with the switch barrier represented as a central peak and the two stable attractors, a differentiated, tumor-suppression state and a self-reinforcing oncogenic state. Across the *x-axis* is an Epigenetic Consensus scale that is essentially a binomial where Pr[Stable] + Pr[Oncogenic] = 1.0 and the threshold transition is defined at Pr[Stable] ≈ Pr[Oncogenic] ≈ 0.50. The *y-axis* is conceived as a instability index gating cellular transition from one state to another. The cross-scale interactions highlighted at the end of Section 3.4 indicate the potential for these scale linkages in epigenetic impacts. Effectively works as three structural lesions each supplying an independent impact against the same central transition, and once that transition is crossed, the population redistributes toward the oncogenic minimum rather than back toward the stable one (the graphical equivalent of the ratchet-like irreversibility envisioned above). The Level 2 columns in Figure 1 are also the conceptual starting point for the analytics proposal below, where each column already enumerates multiple, distinct molecular feature types measured at the same physical scale (for example, four feature classes at the micro scale alone), which is precisely the kind of within-scale evidence that current enrichment methods leave uncombined.

### 4.2. The Approach: Multi-Feature Functional Enrichment

Standard functional enrichment analysis (e.g., Gene Ontology (GO) over-representation testing, Gene Set Enrichment Analysis (GSEA)-style methods [55], or pathway enrichment against KEGG/Reactome) typically takes a single differential feature list as input (for example, genes with altered promoter methylation, or genes with an altered ChIP-seq peak for one histone mark) and tests it for over-representation in annotated gene sets. Each scale, and each molecular feature within a scale, is tested as an independent line of evidence. The cross-scale commonalities argued for above imply that this independence assumption is usually false: because a single underlying regulatory lesion propagates its signature across correlated readouts, within a scale and across scales. Thus, treating those readouts as independent has the effect of discarding true corroboration signals and increasing the background noise (false positives) in our understanding of epigenetic dysregulation.

Two distinct claims must be separated. First, the underlying biology is coupled: a single regulatory lesion propagates its signature across micro-, meso-, and macro-scale readouts, for mechanistic reasons set out in Section 4.1. This coupling makes corroboration expected under the alternative hypothesis and is the reason concordant signals across scales are biologically meaningful. Second, measurement channels are technically distinct: bisulfite or array-based methylation, transposase accessibility, and proximity ligation have largely non-overlapping error models, so an artifact that inflates one channel does not generally inflate the others. This distinctness is what makes corroborative evidence non-redundant (e.g., agreement across channels is more informative than agreement between two replicates of the same assay).

Importantly, the proposal rests on the second claim, not the first, but the second claim itself is not as straight forward as stated above. Multiple assays are typically run on aliquots of the same DNA sample from a tumor specimen and therefore share a dominant set of nuisance factors (shared variances from a common source of origin), mainly tumor purity and stromal/immune admixture, together with somatic copy number, local mappability, GC content, sequencing depth, and processing batch [56,57]. Every one of these can shift all data channels in the same direction. Consequently the channels are correlated under both the null $H_{o}$ as well as under the $H_{a}$ [58,59]. Thus, to effectively integrate data sources with shared within-sample sources of variance, the shared nuisance structure must be modeled and removed before combination. This analytical approach would be ideally calibrated against a null that retains the dependence of channel residuals, such as with randomized permutation test strategies.

#### 4.2.1. Within-Scale Corroboration

Before any within-scale combination, each data channel is adjusted for the shared nuisance structure on a common set of covariates. The key is to avoid methylation-based estimates that would be collinear with the nuisance structure itself [56,60]. Purity deserves particular emphasis here because it is the single largest source of variation in bulk tumor methylation and accessibility data [57], which can vary systematically by tumor type and patient cohort. If this variance is not modeled, it will generate apparent cross-scale concordance at any locus whose signal differs between tumor and stromal compartments. Adjusting this variance reduces the dependence but does not eliminate it (see Section 4.2.3 for handling the residual correlation).

At the micro scale, a candidate promoter methylation change could be corroborated by co-occurring evidence from TET/DNMT sequence-context scores and transcription-factor motif-occupancy change at the same locus. Here these could be combined into a composite per-gene score before enrichment testing. The combination rule must be one that admits dependence. Fisher’s and Stouffer’s methods in their standard form assume independent *p*-values and cannot be directly used here, although their correlation-aware generalizations are useful. Brown’s method extends Fisher’s statistic to dependent tests by approximating $-2\sum \ln p_{i}$ as a scaled chi-square with degrees of freedom set by the covariance of the individual terms. From these approaches, a practical polynomial approximation is available for when only correlations are known [61], and a weighted Stouffer statistic employs this as a correction directly in its denominator [62,63]:

(1) $$Z\;=\;\frac{\sum_{i}w_{i}z_{i}}{\sqrt{\sum_{i}w_{i}^{2}\;+\;2\sum_{i<j}w_{i}w_{j}\rho_{ij}}}$$

In Equation (1), the indices *i* and *j* run over the *k* evidence channels being combined at a locus (here k=3; micro, meso, and macro). The term $z_{i}$ is the signed, covariate-adjusted per-gene statistic from channel *i*, expressed on the standard normal scale, with positive values denoting predicted repression or loss of accessibility. The weight $w_{i}$ is a constant fixed in advance for channel *i*, for example, unity or a value proportional to that channel’s effective sample size. The term $\rho_{ij}$ is the correlation between the statistics of channels *i* and *j* under the null, estimated empirically by permuting sample labels rather than assumed; the summation $\sum_{i<j}$ runs over the k(k−1)/2 distinct channel pairs, and the factor of two restores the symmetric partner of each pair. The denominator is the standard deviation of the numerator, so *Z* is standard normal under the null hypothesis and is directly comparable in scale to any single $z_{i}$. Setting all $\rho_{ij}=0$ recovers the classical Stouffer statistic, making the assumption of independence a special case of Equation (1) rather than a separate method.

At the meso scale, nucleosome-occupancy change (from MNase-seq or ATAC-seq [64]) could be combined with the histone-PTM ChIP-seq signal for the same regulatory element into a composite regulatory-activity score. At the macro scale, Hi-C/TAD-boundary change could be combined with domain-level repressive-mark calls (H3K27me3, H3K9me2) and A/B-compartment switching into a composite domain-disruption score. In each case, the within-scale composite metrics would function like a meta-analysis across distinct molecular readouts of the same physical layer, analogous to combining replicate assays, except, here, the “replicates” are mechanistically distinct feature types predicted (by commonalities 1–4 above) to move together when a locus is genuinely disrupted.

#### 4.2.2. Cross-Scale Integration

A per-gene or per-locus vertically integrated score could then combine the three within-scale composites—micro, meso, and macro—using rank aggregation [65] as a correlation-aware Brown or weighted-Stouffer combination across scales or even as a simple concordant-scale count. The phrase “independent evidence streams” cannot be used at this level because the scales are neither biologically nor technically independent. The cross-scale combinations are more exposed to this problem than the within-scale combinations, because the macro-scale channel contributes a statistic that is shared across every gene in a contact domain. Rank aggregation is comparatively attractive here because it makes no distributional assumption about inputs, but its own significance assessment inherits the same dependence and requires the same empirical calibration [66].

Feeding this cross-scale-corroborated gene list into standard GO, KEGG, Reactome, or GSEA testing [67] would score a pathway as enriched, not merely because many of its genes show some epigenetic change, but because many of its genes show convergent, multi-scale change, directly operationalizing the cross-scale commonalities as a scoring rule rather than a narrative observation. A pathway supported only by macro-scale signal with no micro- or meso-scale corroboration would, under this scheme, be flagged as lower-confidence and a candidate for orthogonal validation, while a pathway with concordant signal across all three scales would be up-weighted for further experiments.

Multi-omics *p*-value fusion methods that combine independent significance estimates across data types before testing pathways do already exist and are in active use [68]. The idea here differs in being motivated by a specific mechanistic prediction, that within-scale features should co-vary because they read out the same underlying lesion (commonalities 1–4), rather than treating cross-platform combination as a purely statistical convenience.

This is a testable hypothesis that can be validated: In public multi-omic cancer cohorts with matched methylation, chromatin-accessibility, and Hi-C data on the same tumors, cross-scale-corroborated gene sets should show improved concordance with known cancer driver pathways and improved reproducibility across independent cohorts, all relative to enrichment run on any single feature type alone. Confirming or falsifying that prediction, rather than the mechanistic synthesis itself, is a concrete next step that is formulated by this perspective.

#### 4.2.3. Calibrating the Null by Permuting Samples Instead of Features

Whichever combination rule is adopted, its null distribution should be obtained empirically by permuting sample labels (e.g., tumor vs normal) and recomputing the entire pipeline, rather than by simply permuting features. Permuting features (shuffling CpGs, peaks, or genes) destroys precisely the structure we are trying to capture, by breaking cross-channel correlation at a locus and block correlation among genes sharing a contact domain. This returns a null distribution that is optimistic by construction [66], because within any single sample all three channels are measured on the same material and therefore carry the same purity, copy-number, and batch signature regardless of which label that sample has been assigned.

There are three implementation points that determine how this works in practice. First, all covariate models must be re-fit inside each permutation because adjusting the observed labels will alter the residuals. Permutations with true residuals in each iteration provide the most unbiased distribution. Second, the permutation scheme must respect the design: matched tumor/normal pairs require within-pair permutation or sign-flipping, not free permutation, which would otherwise produce a null that is conservative in the opposite direction. Third, with the sample sizes realistically available for three-channel matched data, the number of distinct permutations bounds the smallest attainable *p*-value; where the design is too small for adequate resolution, rotation testing can provide a continuous analogue that preserves correlation structure under a linear-model null and is not limited by the number of label assignments [69,70].

The permutation null also supplies a diagnostic worth reporting in its own right. The ratio of the observed test-statistic quantiles to those of the naive independent-combination null can be used as an inflation factor in a sense familiar from genome-wide association studies. This quantifies directly how anti-conservative the independence assumption would have been for a given dataset and a given combination rule. With each channel reduced to one statistic per gene against a common aggregation unit, the null correlation between those statistics is a quantity that can be readily measured by sample-label permutations. Reporting this value, rather than merely employing it as a correction factor, converts an assumption into a diagnostic metric [59]. This value indicates how much of any apparent cross-scale corroboration in a given dataset is just shared nuisance structure rather than hierarchical mechanism.

### 4.3. Testing the Proposal

The Prediction: In cohorts carrying micro-, meso-, and macro-scale measurements on the same tumors, gene sets identified by cross-scale-corroborated scoring will show higher concordance with independently established cancer driver pathways and higher reproducibility across independent contrasts. However, gene sets identified by enrichment on only single features alone (employing equivalent empirically calibrated false discovery rates) will show lower concordance and higher functional divergence. Public multi-omic resources in which two or more scales are profiled on shared specimens already make a version of this test feasible [23].

A Working Hypothetical: Consider a gene $G_{i}$ whose promoter lies inside a repressive domain, and suppose three channels are available: per-CpG methylation at base-pair resolution, chromatin accessibility at nucleosome-to-kilobase resolution, and contact-domain structure at megabase resolution. A regulatory lesion at $G_{i}$ should leave a coordinated signature in all three: hypermethylation across the promoter CpGs, loss of accessibility at the promoter and at the distal elements assigned to it, and altered domain-level context. The proposal focuses on scoring $G_{i}$ using the conjunction of those three signatures that will discriminate the functional relevance more reliably from background variance than scoring $G_{i}$ on any one channel independently.

**Figure 2:**
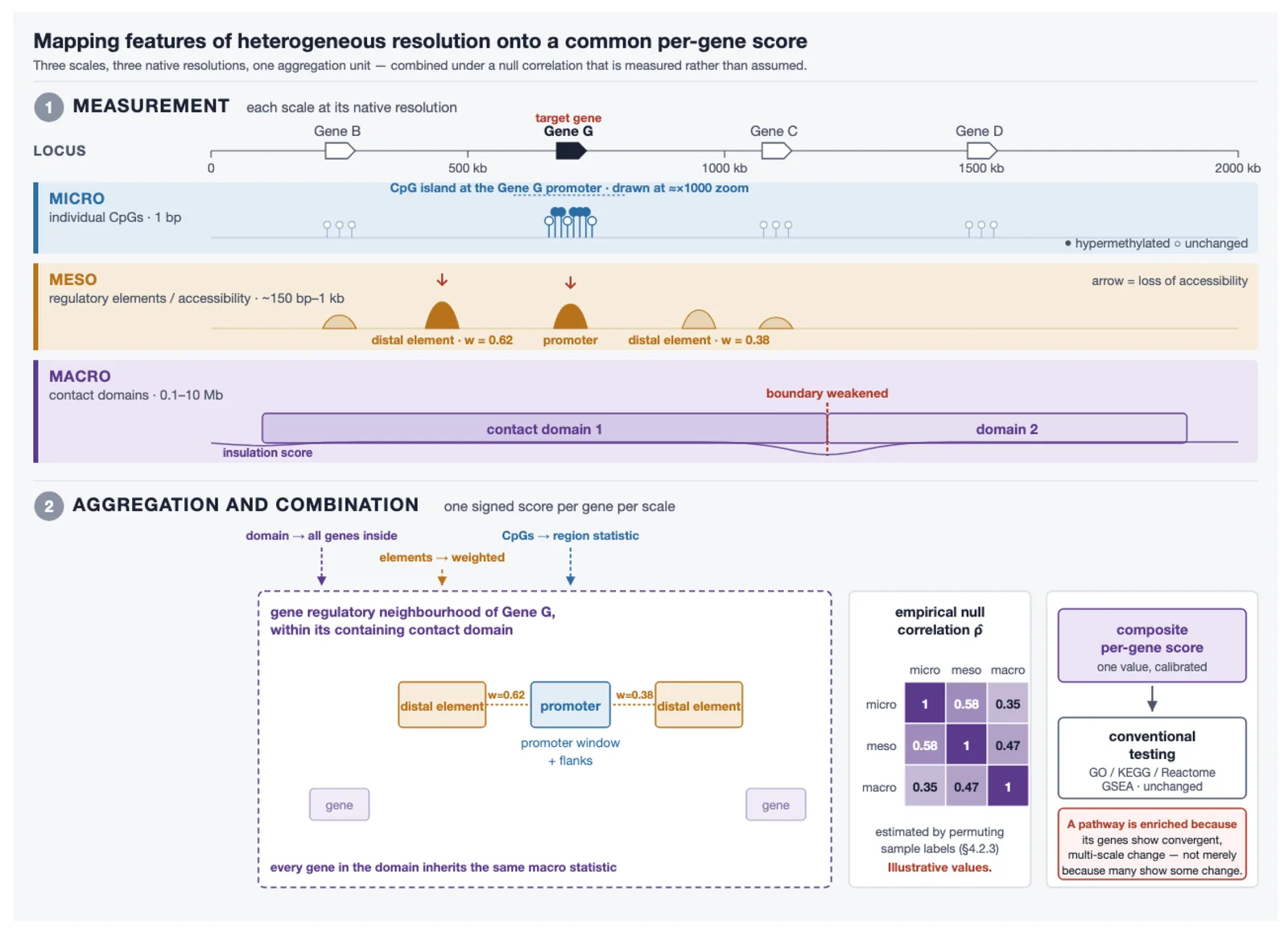
Mapping features of multiple-scales onto a common per-gene score. See Section 4.3.1.

Reducing Multi-Scale Data to One Score: The challenge for integrating diverse channels is that they are not measured in comparable units or at comparable resolutions. The core idea of this proposal is a specific mapping process to reconcile them into one data structure (Figure 2). The unifying construct is a gene regulatory neighborhood where the gene’s promoter window, the distal elements assigned to it, and the contact domain containing it are all balanced to partition nuisance variance away from the main source of variance being analyzed. Each channel is reduced to a single signed statistic per gene against that construct, with the sign fixed by one convention: positive for predicted repression or loss of accessibility. Thus, directional agreement is meaningful across channels physically measuring different quantities:

1. *Micro*: Per-CpG statistics are aggregated over the promoter window and its flanks by a method that models the correlation between neighboring CpGs [71]. Averaging methylation values across a promoter is not adequate: it discards the spatial correlation structure that carries most of the signal.
2. *Meso*: Accessibility changes are computed per regulatory element and assigned to genes by a rule stronger than nearest-neighbor [72], with promoter-proximal and distal contributions kept distinct. An element linking to several genes contributes to each with weights summing to one, so one accessibility change is not counted repeatedly as independent evidence.
3. *Macro*: A gene inherits the domain-level statistics of the contact domain containing its transcription start site.

Every channel is adjusted, before combination, on a common covariate set: tumor purity, copy number, mappability, depth, and batch. Purity (Section 4.2) must come from a channel that is not being corrected, since an estimate derived from methylation is collinear with the micro channel that it would be used to adjust.

#### 4.3.1. The Visual Workflow

In Figure 2, an illustrative workflow for mapping gene and genomic elements across scales is presented. In Panel 1, channel measurements at three scales are aligned against a shared genomic coordinate axis at their native resolutions. Mirco: individual CpGs at base-pair resolution. Meso: regulatory elements and accessibility at nucleosome-to-kilobase resolution with distal elements labeled by the weights through which they are assigned to the target gene. Macro: structural contact domains with an insulation trace at megabase resolution, with one domain boundary marked as weakened. In Panel 2, three aligned cards present the aggregation and combination of target genes. The gene regulatory neighborhood of one is drawn over the same coordinate pattern with promoter window and flanks, assigned distal elements with their link weights, and the containing contact domain. The next card computes an empirical null correlation matrix between the three scale-level statistics, estimated by permuting sample labels and recomputing the pipeline. Finally, the resulting composite per-gene score on the last card enters conventional pathway testing without pipeline modifications.

It is important to note that the Macro scale behaves differently from the other two. The three scales do not enter the composite on equal terms, and the asymmetry is a property of the mapping rather than of the biology. The micro and meso steps contribute statistics computed at or near the gene itself, where a region statistic over that gene’s promoter and accessibility changes at elements are assigned to that gene by weighting coefficients that sum to one. The macro step does something structurally different. It assigns the same domain-level statistic to every gene the domain contains to the target gene and to both of its flanking neighbors alike (see Figure 2).

Gene-level macro scores are therefore block-correlated, over and above the cross-channel correlation discussed in Section 4.2. The effective number of independent units carrying macro-scale evidence is closer to the number of contact domains than to the number of genes. This has practical consequences. First, gene-set tests that treat genes as exchangeable will be miscalibrated on this evidence. A pathway whose members happen to cluster in the genome can be nominated on the strength of what is effectively a single domain-level observation counted once per gene. Second, permuting sample labels leaves the gene-to-domain assignment, and hence the entire block structure, completely intact, whereas permuting features destroys it. Thus, the same permutation scheme that handles the cross-channel dependence also handles the block dependence at no additional cost.

#### 4.3.2. What Would Falsify the Model?

The structural approach of the Model could fail in several ways. First, it would fail if cross-scale composite scoring did not improve the recovery of driver-pathways over the best single channel analysis, with matched empirical false discovery rates. Second, the Model fails if it improves recovery of driver functions but only at a matching cost in label-permuted false discovery. Third, the Model fails if it is unable to recover a known genomic contrast whose cross-scale mechanism is already established (e.g., IDH-mutant glioma chain of Section 3.4). Here, we know TET inhibition produces hypermethylation at insulator sites and consequent domain-level de-repression [25,26]. Thus, there are known cross-scale interactions and linkages that could serve as positive controls spanning the scales this Model is meant to integrate. Any of these outcomes would falsify the analytics proposal while leaving the mechanistic synthesis of Section 2 and Section 3 untouched. That division is deliberate. The underlying synthesis for our Model argument is constructed from published mechanisms across these scales. Thus, the scoring rule proposed here is an empirical and testable claim. However, a failure of the proposed Model would in no way undermine the underlying phenomena of cross-scale corroborative interactions in epigenetic gene regulation which would still remain the benchmark focus for subsequent work.

## 5. Conclusions

Examined together, DNA sequence-level methylation/demethylation targeting, nucleosome occupancy control, and chromosomal domain silencing are not three independent mechanisms but one recurring architecture: a targeted molecular pattern readout followed by cooperative, feedback-driven, spatially bounded spreading, instantiated at three nested physical scales (Figure 1). The three levels developed here are the ones this perspective’s evidence base supports, not a claim that the architecture includes only three. We propose that cancer-associated epigenetic dysregulation is more usefully understood not as a collection of scale-specific lesions but as disruption of this shared logic at any one of its nested levels.

Beyond its explanatory value, this cross-scale architecture argues for a modification in computational practice: functional enrichment statistics should explicitly combine multiple corroborating molecular features, within a scale and across scales, under a null model calibrated to their shared nuisance structure. When testing feature by feature in isolation, that corroborating evidence is not weighed but discarded, and it is precisely this evidence that is most likely to separate a genuine regulatory lesion from a single anomalous measurement. Too often, functional importance in cancer epigenomics is inferred from the methylation status of a single CpG site in a gene promoter. Benchmarking the integrated model in matched multi-omic cancer cohorts would test directly whether multi-feature, cross-scale scoring outperforms conventional single-feature enrichment.

The goal proposed here is a modest one: to improve the confidence that can be placed in functional enrichment analyses of epigenetic oncogenesis. Confidence is what makes a result usable, and trustworthy results are the building blocks from which mechanistic understanding is assembled; a modest gain there propagates into everything built above it.

## Figures and Tables

**Table 1:** Genes and molecular features cited as evidence in Section 2, grouped by physical scale.

ID	Description	Type	References
**Micro scale**			
DNMT3A	De novo DNA methyltransferase; favors a CG(C/T) flanking context	Enzyme (DNA methyltransferase)	[1]
DNMT3B	De novo DNA methyltransferase; favors a CG(G/A) context via TRD-loop residues N779/K777	Enzyme (DNA methyltransferase)	[1,2]
DNMT3L	Catalytically inactive cofactor; flattens the DNMT3A/DNMT3B sequence-preference bias	Cofactor	[3]
TET	Ten-eleven translocation demethylases; sequence-dependent, favor E-box (CACGTG) context	Enzyme (DNA demethylase)	[4,5]
RUNX3	Transcription factor inducing binding-site-directed demethylation	Transcription factor	[6]
GATA2	Transcription factor inducing binding-site-directed demethylation	Transcription factor	[6]
CEBPB	Transcription factor inducing binding-site-directed demethylation	Transcription factor	[6]
MAFB	Transcription factor inducing binding-site-directed demethylation	Transcription factor	[6]
NR4A2	Transcription factor inducing binding-site-directed demethylation	Transcription factor	[6]
MYOD1	Transcription factor inducing binding-site-directed demethylation	Transcription factor	[6]
CEBPA	Transcription factor inducing binding-site-directed demethylation	Transcription factor	[6]
TBX5	Transcription factor inducing binding-site-directed demethylation	Transcription factor	[6]
**Meso scale**			
ISWI	ATP-dependent remodeler family; generates regularly spaced nucleosome arrays	Chromatin remodeler complex	[7,8]
SWI/SNF (BAF)	ATP-dependent remodeler family; reads histone PTMs via bromodomains/PHD fingers	Chromatin remodeler complex	[7,10]
BAF47 (SMARCB1)	BAF subunit required for evicting PRC2/H3K27me3 from tumor-suppressor loci	Chromatin remodeler subunit	[10]
Bromodomain	Reader module recognizing acetylated histone lysines	Reader domain	[7]
PHD finger	Reader module recognizing methylated histone lysines	Reader domain	[7]
Chromodomain	Reader module recognizing methylated lysines	Reader domain	[37,38]
CBX	PRC1 chromodomain subunit family; reads H3K27me3	Reader domain	[34]
H3.3	Replication-independent histone variant; destabilizes nucleosomes	Histone variant	[9]
H2A.Z	Histone variant; combined with H3.3, yields the least stable nucleosomes	Histone variant	[9]
CDKN2A/Ink4a	Tumor-suppressor locus silenced when BAF-mediated PRC2 eviction fails	Tumor-suppressor gene/locus	[10]
**Macro scale**			
PRC2	Polycomb Repressive Complex 2; deposits H3K27me3, spreads via allosteric self-stimulation	Chromatin-modifying complex	[11,12]
PRC1	Polycomb Repressive Complex 1; deposits H2AK119ub, reinforces PRC2 recruitment	Chromatin-modifying complex	[11]
EZH2	PRC2 catalytic subunit; gain-of-function mutation (e.g., Y641) drives oncogenic H3K27 hypermethylation	Enzyme (histone methyltransferase)	[12]
H3K27me3	Repressive histone mark deposited by PRC2	Histone modification	[11,12]
H2AK119ub	Repressive histone mark deposited by PRC1	Histone modification	[11]
LOCKs	Large Organized Chromatin K9-modification domains; expand with differentiation	Chromatin domain	[13]
H3K9me2	Repressive histone mark defining LOCK domains	Histone modification	[13]
CTCF	Boundary protein halting cohesin-mediated loop extrusion at TAD borders	DNA-binding boundary protein	[14,15,16]
Cohesin	Ring complex that processively extrudes chromatin loops	Chromatin-structural complex	[15,16]
TAD	Topologically associating domain; self-interacting, insulated chromatin neighborhood	Chromatin structural domain	[14,17]
A/B compartments	Active/inactive megabase-scale chromatin compartments; conserved across cancer types	Chromatin structural domain	[17]

## Data Availability

No new data were created or analyzed in this study. Data sharing is not applicable to this article.

## Abbreviations

The following abbreviations are used in this manuscript:

A/B compartments	Active (A)/inactive (B) chromatin compartments
ATP	Adenosine triphosphate
BAF	BRG1/BRM-associated factor complex (mammalian SWI/SNF)
BAF47	BAF complex subunit 47 (encoded by *SMARCB1*)
bHLH	Basic helix-loop-helix
bp	Base pair(s)
bZIP	Basic leucine zipper
CBX	Chromobox homolog proteins
CDKN2A	Cyclin-dependent kinase inhibitor 2A
CEBPA	CCAAT/enhancer-binding protein alpha
CEBPB	CCAAT/enhancer-binding protein beta
Chromodomain	chromatin organization modifier domain
CpG	Cytosine-phosphate-guanine dinucleotide
CTCF	CCCTC-binding factor
DNMT3A	DNA methyltransferase 3 alpha
DNMT3B	DNA methyltransferase 3 beta
DNMT3L	DNA methyltransferase 3-like
ES cells	Embryonic stem cells
EZH2	Enhancer of zeste homolog 2
GATA2	GATA-binding protein 2
GO	Gene Ontology
GSEA	Gene Set Enrichment Analysis
H2A.Z	Histone H2A variant Z
H2AK119ub	Histone H2A lysine 119 ubiquitination
H3.3	Histone H3.3 variant
H3K9me2	Histone H3 lysine 9 dimethylation
H3K27me3	Histone H3 lysine 27 trimethylation
HP1	Heterochromatin protein 1
IDH	Isocitrate dehydrogenase
Ink4a	Inhibitor of CDK4a (protein product of *Cdkn2a*)
ISWI	Imitation switch (chromatin remodeler family)
LLPS	Liquid-liquid phase separation
LMO2	LIM domain only 2
LOCKs	Large organized chromatin K9-modifications
MAFB	MAF bZIP transcription factor B
MYOD1	Myogenic differentiation 1
NR4A2	Nuclear receptor subfamily 4 group A member 2
PHD finger	Plant homeodomain finger
PRC1	Polycomb repressive complex 1
PRC2	Polycomb repressive complex 2
PTM	Post-translational modification
RUNX3	Runt-related transcription factor 3
SMARCB1	SWI/SNF-related, matrix-associated, actin-dependent regulator of chromatin, subfamily B, member 1 (BAF47)
SWI/SNF	Switch/sucrose non-fermentable (chromatin remodeling complex)
TAD	Topologically associating domain
TAL1	T-cell acute lymphocytic leukemia protein 1
TBX5	T-box transcription factor 5
TET	Ten-eleven translocation (enzyme family)
TF	Transcription factor
TRD	Target-recognition domain
